# Ability to Participate in Social Activities of Rheumatoid Arthritis Patients Compared with Other Rheumatic Diseases: A Cross-Sectional Observational Study

**DOI:** 10.3390/diagnostics11122258

**Published:** 2021-12-02

**Authors:** Laura Cano-García, Natalia Mena-Vázquez, Sara Manrique-Arija, Rocío Redondo-Rodriguez, Carmen María Romero-Barco, Antonio Fernández-Nebro

**Affiliations:** 1 Instituto de Investigación Biomédica de Málaga (IBIMA), 29010 Málaga, Spain; lauracano.due@gmail.com (L.C.-G.); sarama_82@hotmail.com (S.M.-A.); rocioredondo91@hotmail.com (R.R.-R.); menchu01@hotmail.com (C.M.R.-B.); afnebro@gmail.com (A.F.-N.); 2 UGC de Reumatología, Hospital Regional Universitario de Málaga, 29009 Málaga, Spain; 3 UGC de Reumatología, Hospital Clínico Universitario Virgen de la Victoria, 29010 Málaga, Spain; 4 Departamento de Medicina, Universidad de Málaga, 29010 Málaga, Spain

**Keywords:** rheumatic diseases, rheumatoid arthritis, spondyloarthritis, systemic lupus erythematosus, participate in social activities, psychological factors, mental health

## Abstract

Objectives: To compare the ability to participate in social activities among rheumatoid arthritis patients with other rheumatic disease patients and identify potentially implicated factors. Patients and methods: Between June and November 2019, we consecutively selected patients aged ≥18 years with RA (defined according to ACR/EULAR 2010), SpA (ASAS/EULAR 2010), and SLE (ACR 1997). Main outcome measures: Ability to participate in social roles and activities evaluated using the PROMIS score v2.0 short-form 8a (PROMIS-APS). Secondary outcomes: Participation in social activities according to a series of variables (mobility, depression, satisfaction with social relationships, social isolation, company, emotional support, instrumental support, and support via information). We evaluated the association between the ability to participate in social activities and associated variables using multivariable linear regression analysis. Results: The study population comprised 50 patients with RA (33.1%), 51 patients (33.8%) with SpA, and 50 patients (33.1%) with SLE. The mean PROMIS-APS scores were similar in the three groups. The multivariable analysis for the whole sample showed that the ability to participate in social activities was inversely associated with depression and directly with social satisfaction, mobility, company, and age. The stratified analysis revealed an inverse association between inflammatory activity and ability to participate in social activities in patients with RA and SpA, but not in those with SLE. Conclusion: All patients with RA, SpA, and SLE had a similar ability to participate in social activities. This was associated with other psychosocial factors (social satisfaction, mobility, company, depression) and clinical factors (age and inflammatory activity).

## 1. Introduction

Rheumatic diseases, such as rheumatoid arthritis (RA), spondyloarthritis (SpA), and systemic lupus erythematosus (SLE), are characterized by joint inflammation and other systemic manifestations that lead to functional disability and impaired quality of life [1]. The therapeutic objectives in affected patients include not only reducing inflammatory activity, but also improving quality of life and participation in social activities [2,3]. Previous studies have shown that participation in social activities is closely related to better quality of life and well-being [4].

Patients with rheumatic disease have a reduced capacity for participating in social activities in various settings [5]. Such social isolation may be associated with mood disorders and reduced social satisfaction [6]. Furthermore, the poor visibility of rheumatic symptoms (i.e., pain, stiffness, and fatigue) means that patients are excluded from social exchanges and stigmatized [5,7]. Van Genderen et al. observed that patients with SpA were less satisfied with their interpersonal relationships and leisure interests than controls [8]. Similarly, patients with RA more frequently experience negative sensations with respect to social skills, such as sadness, frustration, and defenselessness [9]. In patients with SLE, loss and uncertainty have been reported to be the two main sensations with respect to social relationships [10]. The first refers to different types of loss—for example, physical ability, appearance, independence, and family balance. In this sense, it is important to note that concerns associated with body image mainly affect self-esteem and social withdrawal in affected patients [11].

However, it is not easy to provide a general measurement of participation in social activities, since this area encompasses a wide variety of domains [12]. One of the most widely used tests for the evaluation of participation in social activities is the Patient-Reported Outcomes Measurement Information Systems (PROMIS) Ability to Participate in Social Roles and Activities v2.0 Short-Form (8 items, PROMIS-APS), which has been shown to have adequate measurement properties [13]. While PROMIS-APS has been used in patients with musculoskeletal disorders [14,15], it has not been specifically evaluated in patients with inflammatory rheumatic diseases. Similarly, no attempts have been made to determine other social factors associated with impaired ability to participate in social activities, since social support is a reasonable treatment goal in interventions that seek to improve psychological well-being in persons with conditions that are associated with disability. Likewise, although these problems are common to all rheumatic diseases, they are experienced differently and affect each type of disease in a different way [16]. In fact, a study comparing social involvement in rheumatic diseases found the greatest degree of involvement in systemic autoimmune diseases such as SLE [5]. However, no comparisons have been made of patients with different inflammatory rheumatic diseases. Therefore, the objectives of our study were to describe the ability to participate in social activities in patients with rheumatoid arthritis compared with SpA and SLE, and likewise to identify the factors involved.

## 2. Materials and Methods

### 2.1. Design

We performed an observational, cross-sectional study of a series of patients with rheumatic diseases (RA, SpA, and SLE). The study was performed at the Instituto de Investigación Biomédica de Málaga (IBIMA) by the Department of Rheumatology of Hospital Regional Universitario de Málaga (HRUM), Malaga, Spain. The study was approved by the Clinical Research Ethics Committee of HRUM (Code no. 2062-N-19).

### 2.2. Patients

We consecutively included patients who visited the rheumatology clinic between June and November 2019. The selection criteria were as follows: age ≥ 18 years, RA according to the 2010 ACR/EULAR classification [17], SpA according to the criteria of ASAS/EULAR 2010 [18], and SLE according to the 1997 criteria of the ACR [19]. Patients were required to have had the disease for more than 24 months. We excluded patients with any other inflammatory or rheumatic disease, patients who could not read or write, and patients with mental disorders that could hamper reading/writing and their understanding of the questionnaire.

### 2.3. Study Protocol

We recruited all patients who fulfilled the inclusion criteria and none of the exclusion criteria. All patients are generally followed prospectively via the rheumatology clinic every 3–6 months according to a pre-established protocol for systematic data collection. The protocol includes variables such as clinical and epidemiological data on disease activity. The reference rheumatologist invited the patients to participate in the study, collected the signed informed consent documents, and recorded the variables in the clinical protocol. The patients then attended the nursing clinic to complete the questionnaires. The nursing department was responsible for explaining the questionnaires to the patients and for resolving any doubts.

### 2.4. Variables and Definitions

The main variable was the ability to participate in social activities, evaluated using PROMIS-APS [20], which comprises 8 questions with 35 items, by means of which we evaluated perceived ability to perform usual social activities in various settings. The scores are numerical and are expressed as T scores, which is a standard scoring system with a mean of 50 and standard deviation (SD) of 10. A higher score indicates a better ability to participate.

The secondary variables evaluated were social activities in terms of various factors, such as mobility, depression, satisfaction with social relationships, social isolation, company, ability to participate in social activities, emotional support, instrumental support, and support via information. All the variables were evaluated using the PROMIS questionnaire with quantitative scores. A higher score indicates increased ability to participate in social activities. All the questionnaires are included in the Supplementary Material.

### 2.5. Other Variables

We also recorded demographic, clinical, and treatment-related data. The demographic and clinical data included age (years), sex, race, educational level (primary, secondary, higher), and socioeconomic level (no income, <€1000 per month, €1000–1500 per month, and >€1500 per month). In addition, we recorded traditional cardiovascular risk factors (current or requiring treatment before data collection, namely, diabetes mellitus, arterial hypertension, dyslipidemia, obesity, body mass index ≥30) and comorbid conditions included in the Charlson comorbidity index [21,22].

The protocol date was the date that the patient was included in the study. Cut-off values were recorded for the Disease Activity Score (DAS) 28 with ESR in RA [23,24], the Bath Ankylosing Spondylitis Disease Activity Index (BASDAI) in spondyloarthritis, and the Safety of Estrogens in Lupus Erythematosus: National Assessment-SLEDAI (SELENA-SLEDAI) in SLE [25].

### 2.6. Statistical Analysis

We performed a descriptive analysis. Qualitative variables were expressed as absolute number and percentage; quantitative variables were expressed as mean (SD) or median and interquartile range (IQR), depending on their distribution. The normality of the continuous variables was confirmed using the Kolmogorov–Smirnov test. The χ^2^ and ANOVA or Kruskal–Wallis test (depending on normality) were used to compare the main characteristics between the 3 groups of patients: (1) patients with RA; (2) patients with SpA; and (3) patients with SLE. Several multivariable linear regression models were then run to assess factors that were independently associated with impaired ability to participate in social activities among all patients with rheumatic disease and in each group individually (continuous dependent variable: ability to participate in social activities).

The multicollinearity of the independent variables was verified using the Pearson correlation coefficient (r > 0.4). The sample size was calculated assuming a prevalence of involvement of 39% in the physical function or pain domain in PROMIS for rheumatic diseases. Considering as relevant a mean of 56.4 with respect to the controls, with a 2-sided α error of 0.05 and β of 0.20, the sample size necessary to detect these differences was 50 patients in each group [26]. Statistical significance was set at *p* < 0.05 for all analyses. All data were analyzed using R 2.4-0.

## 3. Results

We recruited 151 patients between June and November 2019: 50 (33.1%) with RA, 51 (33.8%) with SpA, and 50 (33.1%) with SLE. All the patients completed the questionnaires, and no data were missing.

### 3.1. Patient Characteristics

The main patient characteristics are shown by group in Table 1. Patients with SLE had a mean age of around 47 years, which was slightly lower than those with RA and SpA (*p* = 0.010). There were more women in the RA group (90.0%) and SLE group (96.0%) than in the SpA group (50.0%) (*p* < 0.001). There were no differences in educational level or socioeconomic level between the groups. Most participants had a basic educational level and a socioeconomic level of €1000–1500 per month.

The most frequent comorbid conditions overall were visual impairment (23/151; 15.2%), anxiety (20/151, 13.2%), and depression (according to the clinical history, 21/151, 13.9%). Patients with SLE were more frequently diagnosed with depression (*p* = 0.065) and anxiety (*p* = 0.027) and less frequently with arthritis (*p* < 0.001).

A total of 98/151 patients had a history of peripheral arthritis (64.9%) at the time of the evaluation. The mean DAS28 score in patients with RA indicated low disease activity, the mean BASDAI in patients with SpA was 4, and the mean SLEDAI score was around 5 in patients with SLE. No patients with SpA had psoriasis. The main clinical characteristics of patients with SLE are shown in Supplementary Table S1.

### 3.2. Ability to Participate in Social Activities

The mean PROMIS scores for ability to participate in social activities were very similar between the groups, except for poorer mobility in patients with RA and SpA than in those with SLE (*p* = 0.017) (Table 2).

### 3.3. Factors Associated with Ability to Participate in Social Activities for the Whole Sample of Patients with Rheumatic Disease

Table 3 shows the results of the multivariable linear regression analysis (dependent variable: ability to participate in social activities) for the 151 patients with rheumatic diseases included in the study.

The ability to participate in social activities for all the patients with rheumatic diseases was independently associated with age and the PROMIS items social satisfaction, mobility, depression, and company. Consequently, levels of participation in social activities increased by a mean of 0.07 units per year of age, 0.25 units for each point increase in social satisfaction, 0.15 units for each point increase in mobility, and 0.17 units for each point increase in company; however, the score decreased by 0.17 units for each point increase in depression.

### 3.4. Factors Associated with the Ability to Participate in Social Activities in the Three Groups of Patients

We subsequently ran three separate multivariable linear regression models to identify factors associated with the ability to participate in social activities for each of the groups individually.

Following this approach, we observed that in patients with RA, the ability to participate in social activities was independently associated with satisfaction with social roles, mobility, less social isolation, instrumental support, and lower inflammatory activity according to DAS28 (Table 4). Furthermore, in patients with SpA, the ability to participate in social activities was independently associated with satisfaction with social roles, less frequent depression, company, and lower inflammatory activity according to BASDAI (Table 5). Lastly, in patients with SLE, the ability to participate in social activities was associated with social satisfaction, mobility, and emotional support (Table 6).

## 4. Discussion

Social participation is thought to be a major factor in several health domains (i.e., cardiovascular, neuroendocrine, and immune function) and in the maintenance of health in the general population and in patients with rheumatic diseases [27]. In the general population, reduced ability to participate in social activities is associated with greater mortality, morbidity, and poorer quality of life [28]. We evaluated the ability to participate in social activities (PROMIS-APS) in patients with three rheumatic diseases and its association with other social variables and clinical factors. We found that, despite the epidemiological and clinical differences between the three diseases, abnormalities in patients’ ability to participate in social activities were very similar, and the mean scores of the PROMIS-APS in these three rheumatic diseases were much lower than those of the reference population and patients with other musculoskeletal conditions [29] Likewise, we did not observe differences in the PROMIS score for social activities in the different settings, except for mobility, which was less affected in patients with SLE. The greater impairment in mobility observed in patients with RA and SpA could be associated with older age and more frequent arthritis than in patients with SLE. In this sense, our data are consistent with those of other authors, who report poorer health-related quality of life and physical functioning in patients with RA and SpA than in those with SLE [30,31,32].

On the other hand, we found that improved PROMIS scores for social satisfaction, mobility, company, older age, and lower frequency of depression were independently associated with the ability to participate in social activities (PROMIS-APS). While these aspects have not been evaluated in this type of patient, other studies on patients with musculoskeletal and neuromuscular involvement revealed that participation, as assessed using the PROMIS questionnaire, is associated with better quality of life, greater social satisfaction, and less frequent depression [4,29]. As for age, we found that older patients were more able to participate in social activities. In this sense, whereas adult patients with rheumatic diseases were particularly worried about how their disease could limit their ability to work and their professional goals, adolescents expressed their frustration with differences in the way they were treated by teachers and companions, with the result that this could further affect their participation in social activities [19].

Our study also revealed that participation in social activities by patients with RA and SpA was negatively associated with inflammatory activity, as measured using DAS28 and BASDAI, respectively. This finding is consistent with those of other studies of RA, which found an association between increased inflammatory activity and pain, anxiety, depression, and quality of life [33]. In SpA, inflammatory activity measured using the BASDAI was also associated with greater depression, anxiety, and sleep disorders [3]; therefore, control of inflammation could improve these psychological factors, even participation in social activities. However, this does not seem to be the case in patients with SLE, since we found no association between SLEDAI values and participation in social activities; associations were found only for satisfaction with social roles, mobility, and emotional support. In this sense, one study attempted to evaluate various psychopathological factors and personality dimensions in patients with SLE and found negative correlations for mental health, emotional support, vitality, and perception of general health. Similarly, the authors did not find correlations between any of the clinical variables (SLICC, SLEDAI, or disease duration) and dimensions of quality of life, mental health, and depression [34]. These findings emphasize the importance of analyzing patients’ subjective perceptions of their disease, its impact on their lives, and their symptoms.

Our study was subject to a series of limitations. First, its cross-sectional design prevented us from establishing causality. However, it did enable us to fulfill the primary objective. Second, the PROMIS-APS questionnaire has not yet been validated for rheumatic diseases, although it has proven successful in patients with musculoskeletal conditions; in addition, the reference for PROMIS is the US population and, in our study, was the Spanish population [14,15]. Furthermore, the association between the PROMIS-APS questionnaires and the corresponding scores for participation in different social activities indicates that they are valid instruments. In addition, our study included patients whose understanding and command of the Spanish language was similarly reasonable between the three groups.

## 5. Conclusions

In conclusion, we observed that patients with RA, SpA, and SLE had the same ability to participate in social activities and the three groups of patients had scores below those of the reference population. In addition, we found that the ability to participate in social activities was affected by psychosocial factors (satisfaction, mobility, company, depression) and clinical factors (age and inflammatory activity). It would be interesting to perform interventional studies on these psychosocial factors and inflammatory activity in order to improve participation in social activities and patient quality of life.

## Figures and Tables

**Table 1:** Baseline clinical characteristics of 151 patients with rheumatic diseases.

Variable	RA n = 50	SpA n = 51	SLE n = 50	*p* Value
Epidemiological characteristics				
Female sex, n (%)	45 (90.0)	26 (51.0)	48 (96.0)	<0.001
Age, years, mean (SD)	55.1 (13.6)	52.5 (12.1)	47.1 (11.3)	0.010
Educational level				0.150
None, n (%)	1 (2.0)	0 (0.0)	0 (0.0)	
Primary, n (%)	30 (60.0)	19 (37.3)	21 (42.0)	
Secondary, n (%)	9 (18.0)	18 (35.3)	10 (20.0)	
Higher, n (%)	10 (20.0)	14 (27.5)	19 (38.0)	
Socioeconomic level				0.165
No income, n (%)	1 (2.0)	0 (0.0)	0 (0.0)	
Income <€1000 per month, n (%)	9 (18.0)	6 (11.8)	15 (30.0)	
Income €1000–1500 per month, n (%)	31 (62.0)	30 (58.8)	27 (54.0)	
Income >€1500 per month, n (%)	9 (18.0)	15 (29.4)	8 (16.0)	
Comorbid conditions				
Arthritis, n (%)	50 (100.0)	24 (47.1)	24 (48.0)	<0.001
Osteoporosis, n (%)	9 (18.0)	2 (3.9)	5 (10.0)	0.070
Asthma, n (%)	2 (4.0)	4 (7.8)	1 (2.0)	0.365
COPD, n (%)	1 (2.0)	0 (0.0)	1 (2.0)	0.596
Angina, n (%)	0 (0.0)	1 (2.0)	1 (2.0)	0.605
Heart disease, n (%)	1 (2.0)	3 (5.9)	3 (6.0)	0.556
Acute myocardial infarction, n (%)	0 (0.0)	0 (0.0)	1 (2.0)	0.362
Neurologic disease, n (%)	0 (0.0)	0 (0.0)	0 (0.0)	-
Stroke, n (%)	0 (0.0)	1 (2.0)	3 (6.0)	0.163
Vascular disease, n (%)	0 (0.0)	1 (2.0)	2 (4.0)	0.385
Diabetes mellitus, n (%)	6 (12.0)	3 (5.9)	1 (2.0)	0.128
Gastrointestinal disease, n (%)	5 (10.0)	5 (9.8)	4 (8.0)	0.930
Depression, n (%)	7 (14.0)	3 (5.9)	11 (22.0)	0.065
Anxiety, n (%)	7 (14.0)	2 (3.9)	11 (22.0)	0.027
Visual impairment, n (%)	7 (14.0)	5 (9.8)	11 (22.0)	0.224
Loss of hearing, n (%)	2 (4.0)	8 (15.7)	5 (10.0)	0.146
Vertebral disease, n (%)	5 (10.0)	5 (9.8)	4 (8.0)	0.930
Obesity, n (%)	6 (12.0)	2 (3.9)	5 (10.0)	0.320
Disease activity				
Time since diagnosis in years, mean (SD)	14.3 (7.1)	13.0 (6.1)	18.1 (8.3)	0.593
History of arthritis, n (%)	50 (100.0)	51 (100.0)	24 (48.0)	<0.001
DAS28, mean (SD)	3.1 (1.2)	-	-	-
BASDAI, mean (SD)	-	4.3 (2.1)	-	-
SLEDAI, mean (SD)	-	-	5.3 (4.7)	-

Abbreviations. RA: rheumatoid arthritis; SpA: spondyloarthritis; SLE: systemic lupus erythematosus; COPD: chronic pulmonary obstructive disease, DAS28: Disease Activity Score 28 with ESR; BASDAI: Bath Ankylosing Spondylitis Disease Activity Index; SLEDAI: Systemic Lupus Erythematosus Disease Activity Index.

**Table 2:** Social questionnaires analyzed.

PROMIS	Disease	Mean ± SD	*p* Value
Satisfaction with social roles	RA	25.9 ± 9.1	0.605
	SpA	24.8 ± 9.5	
	SLE	26.6 ± 8.4	
Mobility	RA	48.1 ± 19.3	0.017
	SpA	48.6 ± 17.4	
	SLE	57.1 ± 15.3	
Depression	RA	16.1 ± 7.7	0.937
	SpA	16.6 ± 9.4	
	SLE	16.7 ± 7.9	
Company	RA	16.6 ± 3.8	0.702
	SpA	16.6 ± 4.5	
	SLE	16.6 ± 3.9	
Social isolation	RA	12.4 ± 5.5	0.436
	SpA	13.5 ± 7.4	
	SLE	14.2 ± 7.6	
Emotional support	RA	34.2 ± 7.6	0.706
	SpA	33.5 ± 8.6	
	SLE	32.8 ± 8.5	
Instrumental support	RA	33.1 ± 8.3	0.622
	SpA	31.9 ± 9.0	
	SLE	33.5 ± 8.7	
Support via information	RA	30.4 ± 8.3	0.600
	SpA	32.1 ± 9.0	
	SLE	31.4 ± 8.2	
Ability to participate in social activities	RA	26.2 ± 7.79	0.898
	SpA	26.9 ± 8.2	
	SLE	26.5 ± 6.83	

Abbreviations. RA: rheumatoid arthritis; SpA: spondyloarthritis; SLE: systemic lupus erythematosus.

**Table 3:** Multivariable linear regression analysis of the characteristics associated with ability to participate in social activities in patients with rheumatic disease.

Variable	Univariate OR (95% CI)	ß Regression Coefficient (95% CI)	*p* Value
Age, years	0.034 (0.13, 0.06)	0.070 (0.01, 0.13)	0.021
Female sex	1.346 (−1.65, 4.34)		
Low educational level *	1.037 (−0.34, 2.30)		
Low socioeconomic level **	1.984 (0.12, 3.84)		
Satisfaction with social roles	0.624 (0.53, 0.71)	0.259 (0.13, 0.37)	<0.001
Mobility	0.280 (0.22, 0.33)	0.158 (0.10, 0.21)	<0.001
Depression	−0.599 (−0.70, −0.49)	−0.175 (−0.29, −0.05)	0.004
Company	0.868 (0.60, 1.13)	0.229 (0.01–0.44)	0.038
Social isolation	−0.567 (−0.71, −0.41)		
Emotional support	0.466 (0.34, 0.59)		
Instrumental support	0.300 (0.16, 0.43)		
Support via information	0.504 (0.38, 0.62)		

Nagelkerke R^2^ = 0.49. The variables included in the equation were age, socioeconomic level, satisfaction with social roles, mobility, depression, company, social isolation, emotional support, instrumental support, and support via information. * <€1000 per month. ** None or primary level.

**Table 4:** Multivariable linear regression analysis of the characteristics associated with ability to participate in social activities in patients with rheumatoid arthritis.

Variable	Univariate OR (95% CI)	ß Regression Coefficient (95% CI)	*p* Value
Age, years	0.015 (0.01, 0.15)		
Female sex	7.111 (−0.05, 14.28)		
Educational level	0.483 (−1.83, 2.80)		
Socioeconomic level	3.029 (−0.23, 6.29)		
DAS28	−1.904 (−3.65, −0.15)	−1.251 (−2.27, −0.22)	0.018
Satisfaction with social roles	0.609 (0.43, 0.78)	0.196 (0.01, 0.38)	0.041
Mobility	0.279 (0.19, 0.36)	0.192 (0.11, 0.27)	0.016
Depression	−0.451 (−0.71, −0.19)		
Company	1.027 (0.55, 1.53)		
Social isolation	−0.671 (−0.10, −0.30)	−0.332 (−0.59, −0.06)	0.016
Emotional support	0.392 (0.12, 0.66)		
Instrumental support	0.410 (0.16, 0.65)	0.217 (0.01–0.43)	0.049
Support via information	0.535 (0.30, 0.76)		

Nagelkerke R^2^ = 0.53. The variables included in the equation were age, sex, DAS28, satisfaction with social roles, mobility, depression, company, social isolation, emotional support, instrumental support, and support via information.

**Table 5:** Multivariable linear regression analysis of the characteristics associated with ability to participate in social activities in patients with SpA.

Variable	Univariate OR (95% CI)	ß Regression Coefficient (95% CI)	*p* Value
Age, years	0.039 (0.02, 1.55)		
Female sex	−1.063 (−5.72, 3.59)		
Educational level	2.214 (−0.31, 4.74)		
Socioeconomic level	0.664 (−0.31, 4.44)		
BASDAI	−2.246 (−3.14, −1.34)	−0.768 (−1.39, −0.14)	0.017
Satisfaction with social roles	0.644 (0.48, 0.80)	0.237 (0.05, 0.41)	0.010
Mobility	0.311 (0.21, 0.41)		
Depression	−0.699 (−0.84, −0.51)	−0.439 (−0.61, −0.26)	<0.001
Company	0.653 (0.17, 1.13)	0.301 (0.01–0.58)	0.038
Social isolation	−0.696 (−0.94, −0.45)		
Emotional support	0.503 (0.27, 0.73)		
Instrumental support	0.226 (−0.02, 0.47)		
Support via information	0.506 (0.28, 0.72)		

Nagelkerke R^2^ = 0.51. The variables included in the equation were age, sex, BASDAI, satisfaction with social roles, mobility, depression, company, social isolation, emotional support, instrumental support, and support via information.

**Table 6:** Multivariable linear regression analysis of the characteristics associated with ability to participate in social activities in patients with SLE.

Variable	Univariate OR (95% CI)	ß Regression Coefficient (95% CI)	*p* Value
Age, years	−0.064 (−0.24, 1.20)		
Female sex	4.17 (−5.79, 14.14)		
Educational level	0.547 (−1.52, 2.61)		
Socioeconomic level	2.177 (−0.86, 5.22)		
SLEDAI	−0.125 (−0.54, 0.29)		
Satisfaction with social roles	0.634 (0.48, 0.78)	0.357 (0.20, 0.51)	<0.001
Mobility	0.308 (0.21, 0.40)	0.152 (0.07–0.23)	<0.001
Depression	−0.596 (−0.77, −0.41)		
Company	1.028 (0.60, 1.45)		
Social isolation	−0.407 (−0.63, −0.17)		
Emotional support	0.492 (0.32, 0.66)	0.216 (0.08–0.34)	0.002
Instrumental support	0.295 (0.06, 0.52)		
Support via information	0.472 (0.27, 0.67)		

Nagelkerke R^2^ = 0.47. The variables included in the equation were age, sex, SLEDAI, satisfaction with social roles, mobility, depression, company, social isolation, emotional support, instrumental support, and support via information.

## Data Availability

Data presented in this study are available on request from the corresponding author.

## Supplementary Materials

The following are available online at https://www.mdpi.com/article/10.3390/diagnostics11122258/s1, Table S1: Clinical characteristics of 50 patients with SLE.

## References

[1] García de Vicuña R., Badía B., Garrido E., Prior M. (2010). Artritis reumatoide: Impacto de la enfermedad, análisis de los costes asociados y estudio del acceso a fármacos biológicos en las comunidades autónomas. Rev. Esp. Econ. Salud..

[2] Van Seben R., Smorenburg S.M., Buurman B.M. (2019). A qualitative study of patient-centered goal-setting in geriatric rehabilitation: Patient and professional perspectives. Clin. Rehabil..

[3] Cano-García L., Mena-Vázquez N., Manrique Arija S., Hernández-Sánchez M.D., Segura-Ruiz R., Domínguez-Quesada C., Fernández-Nebro A. (2021). Psychological factors associated with sleep disorders in patients with axial spondyloarthritis or psoriatic arthritis: A multicenter cross-sectional observational study. J. Clin. Nurs..

[4] Douglas H., Georgiou A., Westbrook J. (2017). Social participation as an indicator of successful aging: An overview of concepts and their associations with health. Aust. Health Rev..

[5] Kool M.B., Geenen R. (2012). Loneliness in patients with rheumatic diseases: The significance of invalidation and lack of social support. J. Psychol..

[6] De la Vega R., Molton I.R., Miró J., Smith A.E., Jensen M.P. (2019). Changes in perceived social support predict changes in depressive symptoms in adults with physical disability. Disabil. Health J..

[7] Kool M.B., van Middendorp H., Lumley M.A., Bijlsma J.W., Geenen R. (2013). Social support and invalidation by others contribute uniquely to the understanding of physical and mental health of patients with rheumatic diseases. J. Health Psychol..

[8] Van Genderen S., Plasqui G., van der Heijde D., van Gaalen F., Heuft L., Luime J., Spoorenberg A., Arends S., Lacaille D., Gignac M. (2018). Social Role Participation and Satisfaction with Life: A Study Among Patients with Ankylosing Spondylitis and Population Controls. Arthritis Care Res. (Hoboken).

[9] Fallatah F., Edge D.S. (2015). Social support needs of families: The context of rheumatoid arthritis. Appl. Nurs. Res..

[10] Beckerman N.L., Sarracco M. (2012). Listening to lupus patients and families: Fine tuning the assessment. Soc. Work Health Care.

[11] Zhao Q., Chen H., Yan H., He Y., Zhu L., Fu W., Shen B. (2018). The correlations of psychological status, quality of life, self-esteem, social support and body image disturbance in Chinese patients with Systemic Lupus Erythematosus. Psychol. Health Med..

[12] Eyssen I.C., Steultjens M.P., Dekker J., Terwee C.B. (2011). A systematic review of instruments assessing participation: Challenges in defining participation. Arch. Phys. Med. Rehabil..

[13] Terwee C.B., Crins M.H.P., Boers M., de Vet H.C.W., Roorda L.D. (2019). Validation of two PROMIS item banks for measuring social participation in the Dutch general population. Qual. Life Res..

[14] Liegl G., Rose M., Correia H., Fischer H.F., Kanlidere S., Mierke A., Obbarius A., Nolte S. (2018). An initial psychometric evaluation of the German PROMIS v1.2 Physical Function item bank in patients with a wide range of health conditions. Clin. Rehabil..

[15] Schalet B.D., Kallen M.A., Heinemann A.W., Deutsch A., Cook K.F., Foster L., Cella D. (2018). Using PROMIS Pain Interference Items to Improve Quality Measurement in Inpatient Rehabilitation Facilities. J. Am. Med. Dir. Assoc..

[16] Stamm T., Hieblinger R., Boström C., Mihai C., Birrell F., Thorstensson C., Fialka-Moser V., Meriaux-Kratochvila S., Smolen J., Coenen M. (2014). Similar problem in the activities of daily living but different experience: A qualitative analysis in six rheumatic conditions and eight European countries. Musculoskelet. Care.

[17] Aletaha D., Neogi T., Silman A.J., Funovits J., Felson D.T., Bingham C.O., Birnbaum N.S., Burmester G.R., Bykerk V.P., Cohen M.D. (2010). 2010 Rheumatoid arthritis classification criteria: An American College of Rheumatology/European League Against Rheumatism collaborative initiative. Arthritis Rheum..

[18] Braun J., van den Berg R., Baraliakos X., Boehm H., Burgos-Vargas R., Collantes-Estevez E., Dagfinrud H., Dijkmans B., Dougados M., Emery P. (2011). 2010 update of the ASAS/EULAR recommendations for the management of ankylosing spondylitis. Ann. Rheum. Dis..

[19] Aringer M., Costenbader K., Daikh D., Brinks R., Mosca M., Ramsey-Goldman R., Smolen J.S., Wofsy D., Boumpas D.T., Kamen D.L. (2019). 2019 European League Against Rheumatism/American College of Rheumatology Classification Criteria for Systemic Lupus Erythematosus. Arthritis Rheumatol..

[20] Van der Meij E., Anema J.R., Huirne J.A.F., Terwee C.B. (2018). Using PROMIS for measuring recovery after abdominal surgery: A pilot study. BMC Health Serv. Res..

[21] Gil-Bona J., Sabate A., Miguelena Bovadilla J.M., Adroer R., Koo M., Jaurrieta E. (2010). Charlson index and the surgical risk scale in the analysis of surgical mortality. Cir. Esp..

[22] Charlson M.E., Pompei P., Ales K.L., MacKenzie C.R. (1987). A new method of classifying prognostic comorbidity in longitudinal studies: Development and validation. J. Chronic Dis..

[23] Prevoo M.L., van’t Hof M.A., Kuper H.H., van Leeuwen M.A., van de Putte L.B., van Riel P.L. (1995). Modified disease activity scores that include twenty-eight-joint counts. Development and validation in a prospective longitudinal study of patients with rheumatoid arthritis. Arthritis Rheum..

[24] Esteve-Vives J., Batlle-Gualda E., Reig A. (1993). Spanish version of the Health Assessment Questionnaire: Reliability, validity and transcultural equivalency. Grupo para la Adaptacion del HAQ a la Poblacion Espanola. J. Rheumatol..

[25] Gladman D., Ginzler E., Goldsmith C., Fortin P., Liang M., Urowitz M., Bacon P., Bombardieri S., Hanly J., Hay E. (1996). The development and initial validation of the Systemic Lupus International Collaborating Clinics/American College of Rheumatology damage index for systemic lupus erythematosus. Arthritis Rheum..

[26] Bacalao E.J., Greene G.J., Beaumont J.L., Eisenstein A., Muftic A., Mandelin A.M., Cella D., Ruderman E.M. (2017). Standardizing and personalizing the treat to target (T2T) approach for rheumatoid arthritis using the Patient-Reported Outcomes Measurement Information System (PROMIS): Baseline findings on patient-centered treatment priorities. Clin. Rheumatol..

[27] Mazzoni D., Cicognani E. (2011). Social support and health in patients with systemic lupus erythematosus: A literature review. Lupus.

[28] Levasseur M., Desrosiers J., St-Cyr T.D. (2007). Comparing the Disability Creation Process and International Classification of Functioning, Disability and Health models. Can. J. Occup. Ther..

[29] Tamminga S.J., van Vree F.M., Volker G., Roorda L.D., Terwee C.B., Goossens P.H., Vliet Vlieland T.P.M. (2020). Changes in the ability to participate in and satisfaction with social roles and activities in patients in outpatient rehabilitation. J. Patient Rep. Outcomes.

[30] Chen H.H., Chen D.Y., Chen Y.M., Lai K.L. (2017). Health-related quality of life and utility: Comparison of ankylosing spondylitis, rheumatoid arthritis, and systemic lupus erythematosus patients in Taiwan. Clin. Rheumatol..

[31] Benitha R., Tikly M. (2007). Functional disability and health-related quality of life in South Africans with rheumatoid arthritis and systemic lupus erythematosus. Clin. Rheumatol..

[32] Ovayolu N., Ovayolu O., Karadag G. (2011). Health-related quality of life in ankylosing spondylitis, fibromyalgia syndrome, and rheumatoid arthritis: A comparison with a selected sample of healthy ındividuals. Clin. Rheumatol..

[33] Vergne-Salle P., Pouplin S., Trouvin A.P., Bera-Louville A., Soubrier M., Richez C., Javier R.M., Perrot S., Bertin P. (2020). The burden of pain in rheumatoid arthritis: Impact of disease activity and psychological factors. Eur. J. Pain.

[34] Barbosa F., Mota C., Patrício P., Alcântara C., Ferreira C., Barbosa A. (2011). The relationship between alexithymia and psychological factors in systemic lupus erythematosus. Compr. Psychiatry.

