# Supplementary material for: Ability to Participate in Social Activities of Rheumatoid Arthritis Patients Compared with Other Rheumatic Diseases: A Cross-Sectional Observational Study
Source: Diagnostics (Basel). 2021 Dec 2;11(12):2258. doi: 10.3390/diagnostics11122258 (PMC8700244; doi:10.3390/diagnostics11122258)
Supplement: Supplementary file 1 [file diagnostics-11-02258-s001.zip › diagnostics-1479513-supplementary.pdf]

Table S1: Clinical characteristics of 50 patients with SLE.

Variable	SLE (n=50)
Epidemiological characteristics	
Sex (female), n (%)	48 (96.0)
Age, years, mean (SD)	47.1 (11.3)
Time since diagnosis in years, mean (SD)	18.1 (8.3)
Clinical manifestations	
Malar rash, n (%)	31 (62.0)
Photosensitivity, n (%)	38 (77.8)
Ulcer, n (%)	30 (60.3)
Arthritis, n (%)	24 (48.0)
Serositis*, n (%)	18 (36.1)
Neurologic**, n (%)	1 (2.0)
Haemolytic***, n (%)	16 (32.0)
Nephritis, n (%)	17 (33.3)
Interstitial lung disease, n (%)	0 (0.0)

Abbreviations. SLE: systemic lupus erythematosus; *SD*, standard deviation; *serositis: pleuritis and pericarditis; **Neurologic: seizure and psychosis; ***Hematologic: haemolytic anaemia, leukopoenia and thrombocytopenia
